# From Mother to Infant, from Placenta to Gut: Understanding Varied Microbiome Profiles in Neonates

**DOI:** 10.3390/metabo13121184

**Published:** 2023-12-05

**Authors:** Riadh Cheddadi, Venkata Yeramilli, Colin Martin

**Affiliations:** Department of Surgery, Division of Pediatric Surgery, Washington University School of Medicine, Saint Louis, MO 63110, USAcolinm@wustl.edu (C.M.)

**Keywords:** neonatal microbiome, metabolites, maternal microbiome, microbiome transfer, maternal influence, omics, animal models, pregnancy

## Abstract

The field of human microbiome and gut microbial diversity research has witnessed a profound transformation, driven by advances in omics technologies. These advancements have unveiled essential connections between microbiome alterations and severe conditions, prompting the development of new frameworks through epidemiological studies. Traditionally, it was believed that each individual harbored unique microbial communities acquired early in life, evolving over the course of their lifetime, with little acknowledgment of any prenatal microbial development, but recent research challenges this belief. The neonatal microbiome’s onset, influenced by factors like delivery mode and maternal health, remains a subject of intense debate, hinting at potential intrauterine microbial processes. In-depth research reveals associations between microbiome profiles and specific health outcomes, ranging from obesity to neurodevelopmental disorders. Understanding these diverse microbiome profiles is essential for unraveling the intricate relationships between the microbiome and health outcomes.

## 1. Introduction

The field of human microbiome and gut microbial diversity research has undergone significant transformation in response to the rapid advancement of omics technologies. While various aspects of biological research have experienced considerable progress, the trajectory of microbiome investigations is distinctive. This transformation involves a transition from culture-based approaches for analyzing oral microbial composition to molecular techniques for identifying microbial profiles across diverse ecological niches within the human body [1,2]. These advancements have revealed crucial associations between microbiome alterations and severe conditions spanning from neuropsychiatric disorders to cancer. Consequently, the development of new frameworks and model systems has been facilitated by microbiome epidemiological studies [3,4]. As an illustration, a notable example is the demonstrated influence of the gut microbiome on modulating the response of melanoma to anti-programmed cell death 1 protein (PD-1) immunotherapy [5].

The prevailing notion was that each human harbored predominantly unique microbial communities, which were traditionally believed to be acquired in early life; this phenomenon has been the cornerstone of numerous surprises in both basic scientific research and translational applications, giving rise to multiple discoveries and an abundance of answers [6,7]. One highly debated facet of the microbiome is its temporal dynamics. Some advocate for the long-term stability of the human gut microbiota [8], while others illustrate a fluctuating array of microbiome profiles across various life stages [9]. A more plausible framework involves embracing both viewpoints, considering the microbiome as a persistent profile with relatively rapid transitions during specific critical periods. Microbial diversity varies significantly across diverse ecological niches within the adult human body. For example, the gut is renowned for its notably high microbial diversity, which can be disrupted by various conditions such as necrotizing enterocolitis and inflammatory bowel disease [10]. In order to gain a comprehensive understanding of the natural progression of the microbiome, it is imperative to focus on the early neonatal stages and the numerous factors contributing to microbial variations. Factors such as breastfeeding, antibiotic usage, environmental contaminants, and nutritional status play pivotal roles. From the very beginning, the neonatal oral cavity encounters a diverse array of microorganisms, and the initial set of colonizers naturally leads to subsequent colonization patterns [11]. An illustrative example of a colonizer is the presence of Gram-negative bacteria and the subsequent production of short-chain fatty acids (SCFAs). This sequence of events can lead to a colonization pattern characterized by the disruption of the intestinal barrier and an increase in intestinal permeability to bacterial toxic metabolites [12,13].

The impact of pregnancy on the infant microbiome has been the subject of extensive discourse in recent years. Despite evidence indicating the absence of viable bacteria in the fetus [14], there remains a lack of consensus regarding intrauterine colonization. Nevertheless, a noteworthy observation has highlighted differences in microbial profiles among neonates immediately after birth, suggesting the potential existence of an intrauterine microbial process [15]. In light of these considerations, the conventional belief in a post-birth or early-life acquired microbiome has come under scrutiny. This raises the question of how two neonates born at the same gestational age can possess different initial microbial profiles. 

## 2. The Neonatal Microbiome

The human gut microbiome exhibits remarkable richness and harbors dynamic populations of microorganisms, primarily characterized by the dominance of bacterial communities. This ecosystem comprises approximately 3.8 × 10^13^ cells, collectively bearing a genome that surpasses the human genome by approximately 150-fold [16]. The multitude of trillions of cells residing within the gut represents the most abundant microbial population, and their pivotal role in host development and overall health is mediated via direct interactions with the host or through the influence of various metabolites. These interactions occur within the context of a highly homeostatic ecosystem, often referred to as the host–symbiont or holobiont. This conceptual framework acknowledges the integral role of coevolution in shaping the composition of the gut microbiome and its impact on human development. Consequently, any disruption in the long- or short-term selective pressures acting on the microbiome is bound to have significant consequences for neonatal development [17,18,19].

**Figure 1:**
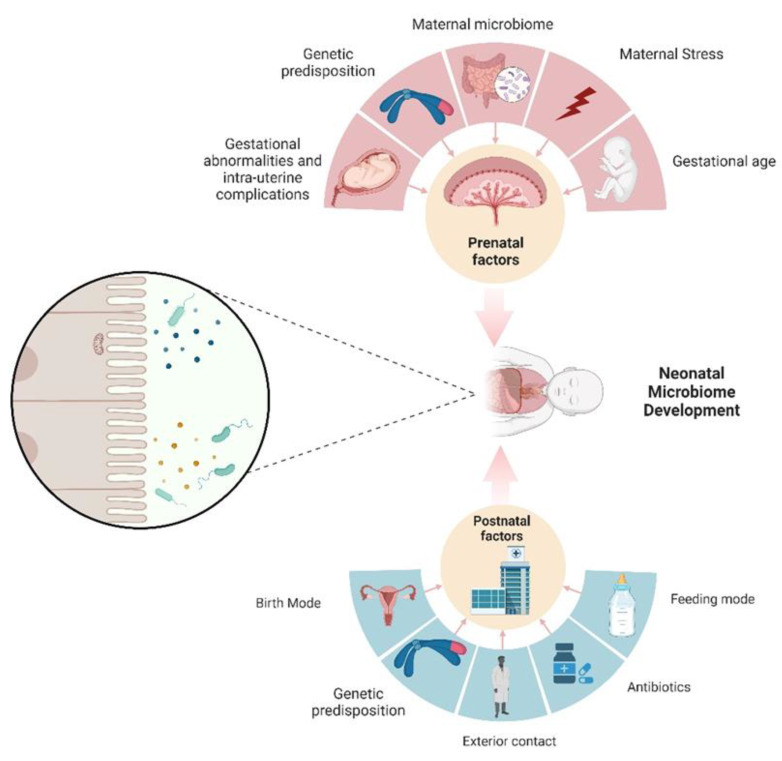
An infant’s microbiome undergoes significant changes, both in terms of quantity and quality, due to various influences. These factors can be categorized into two main groups: those that occur before or after birth. Notably, the mother has a substantial impact on the prenatal development of the neonatal microbiome, and this influence persists even after birth through different mechanisms.

The objective of this article is to establish a consensus regarding the onset of neonatal microbiome development (Figure 1). Historically, the prevailing view has held that the intrauterine environment is devoid of microorganisms, with gut colonization commencing only at the moment of birth [20]. Within this paradigm, researchers who have investigated the post-birth neonatal microbiome have portrayed it as an initial set of microbial sets that undergo maturation and progression into a more intricate microbiome, characterized by an enrichment of *Bacteroides* and *Firmicutes*, which are representative of an adult-like microbiome [21]. Within this perspective, the colonization of the neonatal gut constitutes a de novo construction of a microbial community and is influenced by a multitude of factors. These factors include considerations such as age, dietary regimen, method of delivery, concurrent health conditions, antibiotic usage, and the birthing environment of the infant (NICU) [22,23]. The relationship between neonatal age and the mode of birth presents an intriguing connection, given that a significant proportion of premature infants are delivered via a C-section [24]. It is important to note that the microbiota of preterm infants are observed to contribute to the maintenance of an already fragile innate immune system. Consequently, any aberrant colonization of the gut microbiota may lead to unfavorable outcomes [25].

To challenge this post-birth colonization theory, others have demonstrated a different set of arguments. Meconium, the initial intestinal discharge in newborns, is a substance that primarily consists of materials ingested rather than secreted by the gastrointestinal tract during the intrauterine period. Conventionally, both amniotic fluid and meconium have been regarded as sterile under normal circumstances. This paradigm held validity because attempts were made to culture bacteria that were largely non-culturable. However, with the advent of molecular techniques, particularly those based on 16S rRNA, microorganisms have been identified in both meconium and amniotic fluid [26]. High-throughput meconial microbial sequencing has revealed a substantially distinct microbial taxonomy compared to that which could be attributed to potential cross-contamination from the anus or uterus. This finding implies the possibility of intrauterine seeding and colonization of the neonatal gut [27]. Others have approached this theory from a different perspective, as demonstrated by Warner et al. [28]. They collected 3586 stool samples from 166 infants in two distinct cohorts and identified a unique microbial profile that low-birth-weight infants exhibit immediately after birth. Specifically, they observed a predominance of *Gammaproteobacteria* and a relative scarcity of strict anaerobic bacteria, which preceded the onset of necrotizing enterocolitis [15]. This research not only illustrated the pre-birth origin of the neonatal microbiome but also revealed the association between dysbiosis and a devastating condition, necrotizing enterocolitis.

## 3. Microbial Transfer and Postnatal Influences

The concept of a placental microbiome remains “again” a subject of considerable debate [28]. Aagaard et al. reported the presence of a microbiome in the placenta, resembling that of the oral cavity, under sterile conditions [29]. Subsequent investigators have corroborated Aagaard’s findings and argued that if there is a microbiome in blood and other human niches previously deemed sterile, it logically follows that there should be a distinct microbiome in the placenta as well [29,30]. Another intriguing discovery from the same research project involved the investigation of a potential connection between specific placental microbiome types and neonatal outcomes. They identified distinct placental taxa that correlated with preterm birth, shedding light on a potential link between the placental microbiome and adverse pregnancy outcomes [29]. Conversely, other investigators have questioned this entity, and argued that the human placenta does not have a microbiome but it does represent a potential site for microbial acquisition. De Goffau et al. employed distinct methods for DNA extraction and detection, and they based their conclusions on several factors. One key factor was the notably low bacterial sequence biomass in DNA extracted from the placenta, with a significant portion attributed to potential contamination during labor, delivery, or laboratory processes [31,32]. From our perspective, some of the most compelling evidence supports the existence of a placental microbiome, as indicated by the identification of a microbiome in fetal meconium, as extensively discussed previously. This assertion is further confirmed by animal model studies that consistently yield reproducible evidence of cultivatable fetal bacteria [33]. However, it is crucial to acknowledge that the majority of the analytical tools employed, such as a 16S rRNA analysis, are qualitative rather than quantitative. This implies that these findings underscore the presence of specific taxa without providing quantitative information about the amount of bacteria present.

The transmission of specific bacterial taxa is heavily dependent on the mode of birth. Infants either inherit a microbial package resembling that found in the mother’s birth canal, or this maternal–fetal microbial overlap is lost when neonates are delivered via a cesarean section [34]. In the case of a cesarean section, other factors appear to have a more significant influence on the postnatal microbiome as compared to the prenatal microbiome. These factors primarily include environmental elements such as delivery and surgical equipment, healthcare workers, and contact with other neonates [22]. *Lactobacillus* appears to be a prominent feature of vaginal delivery, being highly abundant, particularly in the maternal vagina. Infants born via cesarean section exhibited a consistently low detection rate of *Lactobacilli* for up to 6 months after birth. Surprisingly, this disparity in bacterial taxa disappears by the time the infants reach three years of age [35].

On the immediate postnatal aspect, milk is recognized as the primary exogenous source of nutrition for the newborn [36]. Consequently, the composition of milk is believed to play a crucial role in shaping the microbial composition of the infant [37,38]. The composition of milk is subject to changes during the lactation process and can also differ from one mother to another. Apart from the well-documented increase in protein levels in the milk of mothers who delivered preterm infants [39], variations in the maternal milk microbiome are observed among individuals with different lifestyles and backgrounds. For instance, there is a higher diversity in microbial taxa noted in mothers with urban lifestyles compared to those with more rural lifestyles [40,41]. In situations where maternal milk is not available, artificial milk formula serves as the primary alternative. Despite substantial scientific advancements in its production to closely mimic authentic breast milk, significant disparities still exist [42]. From a microbial perspective, formula lacks the diverse bacterial communities essential for immune adaptation and the healthy development of the gut, as naturally found in breast milk [43]. Even with the introduction of prebiotic-supplemented formula, it is important to note that breast milk has been shown to be uniquely tailored to each newborn, with predetermined quantities and qualities of bacterial taxa influenced by the mode of birth and genetic backgrounds [44]. 

Similarly, medication, particularly antibiotics, has been implicated in causing deleterious effects on the human gut microbiome, particularly when administered during the early neonatal phase [45,46,47]. The microbial alterations induced by antibiotics are deemed to have adverse implications for the well-being and prospective development of newborns. Descriptively, antibiotic-triggered modifications to the microbiome appear to affect distinct facets, specifically, diversity, temporal stability, and quantitative distribution [48].

Environmental factors have also been documented to exert an influence on the neonatal microbiome. Studies have revealed disparities, particularly in terms of both the diversity and predominant bacterial types, contingent upon the birthplace. At the compositional level, infants born in hospital settings tend to exhibit lower levels of *Bacteroides*, *Bifidobacterium*, *Streptococcus*, and *Lactobacillus*, while displaying higher proportions of the *Clostridium* and *Enterobacteriaceae* families compared to infants born at home [49]. Notably, these disparities are reflective of a proinflammatory phenotype, marked by an overexpression of various inflammatory markers in infants born in hospital environments [50]. It is essential to emphasize, nevertheless, that some studies have demonstrated that the place of birth plays a role in the divergence of an initially similar microbiome. In other words, the initial bacterial colonization does not differ significantly between the two groups [51].

## 4. Prenatal Influences on Neonatal Microbiome

The concept of Developmental Origins of Health and Disease (DOHaD) encompasses a field of investigations proposing that detrimental exposures occurring in the early stages of life, while tissues and organs are undergoing development, may elevate the susceptibility to diseases later in life [52,53]. The majority of embryonic development and organogenesis happens during the intrauterine period, where the fertilized oocyte develops into a coordinated assembly of interconnected organs. In alignment with this paradigm, compelling epidemiological evidence and experimental data in animal models have substantiated a robust association between the intrauterine environment, often represented by the maternal factors, and the subsequent risk of infants developing diseases in later stages of life [54,55].

The concept of allostatic load finds its manifestation in the context of pregnancy, which can be regarded as a unique state of “allostasis” wherein the maternal–fetal dyad faces the intricate challenge of combining the dual objectives of maternal and fetal well-being across the course of development [56]. Maternal adversity experienced during pregnancy can be predominantly associated with environmental factors. The spectrum of maternal stressors is expansive and profoundly impactful, including factors such as partner violence, healthcare accessibility, housing conditions, experiences of humiliation, racial discrimination, and a heightened sense of danger. Many of these stressors can be comprehensively categorized under the broader umbrella of social determinants of health [57]. The mechanisms through which maternal adversity can influence microbiome development are interconnected with dysregulation of the hypothalamo-pituitary axis, pronounced cytokine secretion, direct placental effects, and metabolic alterations [58]. In a rodent model assessing stress during pregnancy, it has been illustrated that maternal stress exerts an influence on the postnatal colonic microbiome, manifesting from postnatal day 2 through postnatal day 28. Notably, a disrupted microbiome structure was detected in male offspring as they displayed characteristics akin to the microbiome patterns typically associated with females, thus suggesting a linkage between stress, hormonal factors, and the gut microbiome [59]. From a descriptive perspective, the stress-induced alterations in the microbiome encompassed a concurrent proliferation of facultative anaerobic microorganisms at the detriment of obligate anaerobes [60,61]. This observation is noteworthy, as it implies that specific taxa of facultative anaerobes, such as *Mucispirillum* and *Desulfovibrionaceae*, collectively possess the capacity for mucin degradation and the production of hydrogen sulfide [52]. These changes are regarded as a plausible foundation for the initiation of intestinal inflammation [62,63].

Studies involving site- and strain-specific gut microbiota profiling have illuminated the influence of the maternal genotype on the fetal and neonatal microbiome. These investigations reveal a complex interplay between the maternal environment and genetic background in shaping the microbiome of neonatal offspring. Notably, murine models have proven the presence of a novel maternal effect introduced by the birth mother. Researchers have employed techniques such as the transplantation of pups to dams with differing genetic backgrounds to disentangle the influence of microbiota from their respective families. Within the framework of these studies, it was observed that mice from distinct genetic lines, when born together after transplantation to a new dam, displayed similar microbiota profiles [64]. Human studies have bolstered this line of thinking, particularly through twin studies, and have consistently affirmed the influence of host genetics on microbial assemblages. Notably, examinations of monozygotic and dizygotic twins have provided compelling evidence. These investigations have consistently shown that neonates born from monozygotic twins tend to manifest greater similarity in their gut microbiota profiles compared to their counterparts born from dizygotic twins [65,66].

Maternal dietary patterns have the potential to impact the developing fetus through multiple mechanisms [67], and it is widely hypothesized that one of these mechanisms involves influencing the composition of the neonatal microbiome. A study investigating the microbial DNA in the amniotic fluid and placenta from pregnant mothers administered probiotic compounds and revealed a significant alteration in innate immunity gene patterns [68]. In this direction, specific probiotic bacterial-derived metabolites show promise as potential perinatal therapeutic interventions [69]. Numerous medications have been identified as having adverse effects on fetal development [70]. Nonetheless, only a limited number of drugs have been recognized for their ability to mitigate certain effects through the modulation of the maternal and fetal microbiome, with antibiotics being one notable example. In animal models, when administered to pregnant dams, it was noted that there were significant changes in the neonatal gut microbiome, a reduction in intestinal host defense, and an elevated risk of neonatal sepsis. These observations were attributed to a potential decrease in the transmission of bacteria during or shortly after delivery. It is also plausible, however, that maternal antibiotics may limit the colonization of the fetal gut by bacteria even before birth, leading to an atypical immune priming [71].

## 5. Interpreting Varied Microbiome Profiles

Up to this point, we have discussed the factors contributing to a variable microbiome in neonates. The continuity involves pinpointing the relationships between specific microbiome taxa and particular health outcomes. It is imperative to acknowledge that an infant’s microbiota are inherently distinguished by lower bacterial abundance and diversity. As the infant matures, the microbiota progressively become more complex. Analogously, the rudimentary, less diverse microbiota in early infancy can be likened to a solid foundation for a building. Should this foundation be flawed, any structure constructed upon it is predisposed to instability and eventual deterioration [72,73,74]. Considering this perspective, particular microbiome profiles can be directly associated with specific diseases. In-depth research has demonstrated that disruptions in early-life microbiota are conducive to the development of obesity induced by a high-fat diet; further investigation showed that these alterations are primarily instigated by the depletion of *Lactobacillus* species within the gut microbiota [53]. In the same experimental model, it elucidated the decrease in Lactobacillus-derived metabolites, particularly phenyllactic acid, known to activate peroxisome proliferator-activated receptor γ (PPAR-γ), a key regulator of lipid metabolism [75]. Another illustration of how the microbiome engages with the immune system involves molecular signaling and the participation of innate immunity, facilitated by various microbial species and microbiome-related molecules. The colonization of the gut by Gram-negative bacteria that secrete lipopolysaccharides (LPSs) has been identified as a pivotal factor in this mechanism. LPS serves as the trigger for initiating inflammatory responses, particularly those mediated through Toll-like receptor 4 (TLR-4) and nuclear factor kappa B (NF-κB) [76,77,78].

Extensive research efforts have been dedicated to exploring the influence of the microbiome on neurodevelopmental and neuropsychiatric disorders, which has paved the way for the emerging concept of the gut–brain axis [79,80,81]. The communication between the microbiota and the brain occurs through multiple pathways, including interactions with the immune system, modulation of tryptophan metabolism, involvement of the vagus nerve, and the enteric nervous system. This also includes the influence of microbial metabolites such as short-chain fatty acids, branched-chain amino acids, and peptidoglycans [82]. A specific neurodevelopmental outcome that has accumulated significant attention in microbiome-related research is autism spectrum disorder (ASD) [83,84,85,86,87]. ASD is frequently accompanied by concurrent dysbiosis, which gives rise to gastrointestinal symptoms like motility problems and abdominal pain [88]. In the past, these gastrointestinal symptoms were perceived as entirely unrelated; however, as our understanding of ASD deepens, it becomes increasingly evident that these gastrointestinal symptoms often correlate with the severity of behavioral differences in individuals with ASD [89,90]. In both fecal samples from individuals with ASD and mouse models exhibiting ASD, there has been a notable increase in the shared presence of certain genera. Specifically, an elevated abundance of *Bilophila*, *Clostridium*, *Dorea*, and *Lactobacillus*, coupled with a concurrent decrease in the *Blautia* genera, has emerged as particularly relevant to this disorder [87].

## 6. Case Studies and Animal Models

Throughout this review, we have highlighted the critical importance of the chronological sequence of events in microbiome development, underscoring the need for longitudinal studies of the microbiota across different age groups. Microbiome research employing animal models has primarily focused on the use of mice [91] and Drosophila melanogaster [92,93]. Nevertheless, we estimate that significant contributions to our understanding of microbiome research can be achieved by utilizing simpler animal models characterized by lower taxonomic diversity. Invertebrate models facilitate cost-effective longitudinal studies of the microbiota across shorter timescales. Invertebrate models offer the advantage of enabling complex experimental designs while simultaneously circumventing ethical concerns related to research on mammals. One such model is the short-lived nematode, *Caenorhabditis elegans*. The utilization of this model is greatly facilitated by the wealth of available resources and the extensive availability of mutants, particularly through the Caenorhabditis Genetics Center (CGC) [94]. *C. elegans* possesses a significant advantage due to its transparency, which allows for the straightforward visualization of fluorescently labeled microorganisms within its gut. This transparency also eases the real-time tracking of the spatiotemporal distribution of gut bacteria. It is worth noting that *C. elegans* is a microbivore, and its laboratory maintenance involves feeding it *Escherichia coli OP50*. To mitigate the impact of this exogenous administration of bacteria, one approach is to treat the *E. coli* with UV or heat to render it nonviable [95]. From an anatomical perspective, the intestine represents the largest somatic organ in the worm and typically serves as a habitat for a diverse array of microorganisms [96]. The *C. elegans* intestine is succinctly characterized by a continuous monolayer of 20 non-renewable epithelial cells, collectively forming a tubular structure with a central lumen [97]. Practically, in *C. elegans*, the inclusion of live probiotic bacteria, such as *Lactobacillus* and *Bifidobacterium*, has been shown to boost immune defenses and extend the organism’s lifespan. Consequently, *C. elegans* can serve not only as a valuable model for microbiome research but also as a tool for exploring dietary interventions involving probiotics [98].

## 7. Conclusions

In conclusion, the field of human microbiome research has evolved significantly, driven by the advent of omics technologies. It has revealed the intricate associations between microbial alterations and various health conditions. While the prevailing belief once maintained that each individual housed unique microbial communities primarily acquired and evolving throughout their lifetime, without acknowledging a prenatal source, recent research has challenged this notion. The neonatal microbiome development, including factors such as mode of delivery, maternal health, and postnatal influences, remains a subject of intense scrutiny and debate. Evidence suggesting the possibility of intrauterine microbial processes and the influence of prenatal factors on the neonatal microbiome challenges the traditional post-birth acquisition theory. The microbiome’s impact on various aspects of health, including its role in neurodevelopmental disorders like autism spectrum disorder, underscores the need for further research and exploration. Utilizing animal models, such as *Caenorhabditis elegans*, facilitates microbiome investigations and offers insights into dietary interventions with probiotics. Understanding these diverse microbiome profiles is crucial in unraveling the complex relationships between the microbiome and health outcomes.

## 8. Future Directions

An extensive body of research has been dedicated to the analysis of microbiome composition, with recent groundbreaking revelations largely driven by a 16S rRNA analysis [15]. However, in this field, it is notable that productivity has appeared to plateau, as much of the recent work is primarily focused on reaffirming established facts or rectifying previously held paradigms. A promising avenue for further exploration lies in the realm of bacteriophages [99], which are viruses that infect prokaryotic organisms. These entities have been identified wherever bacterial hosts are present, and akin to their bacterial counterparts, bacteriophage communities may be associated with various health and disease states. While it is evident that a bacteriophage analysis could offer valuable insights, accessing this information remains challenging due to the substantial presence of temperate phages, which can stably reside within bacterial genomes. Additionally, the absence of a distinctive marker gene, analogous to the 16S rRNA in bacteria, further challenges such analyses [100]. Although these challenges complicate our comprehension of disease-driving bacteriophage-mediated mechanisms, they also present an opportunity for the discovery of biomarkers linked to gut microbial dysbiosis. Furthermore, the exploration of fungi and viruses and their intricate interactions with gut microbial entities remains an area with limited understanding [101,102]. It is our assessment that a more inclusive and comprehensive analysis, encompassing both bacterial and non-bacterial components, holds significant importance in delineating various neonatal microbiome profiles.

## Data Availability

The authors confirm that the data supporting the findings of this study are available within the article.
